# From skepticism to assurance and control; Implementation of a patient safety system at a pediatric hospital in Sweden

**DOI:** 10.1371/journal.pone.0207744

**Published:** 2018-11-26

**Authors:** Ann-Charlotte Almblad, Mats Målqvist, Gunn Engvall

**Affiliations:** Department of Women’s and Children’s Health, Uppsala University, Uppsala, Sweden; Akershus University Hospital, NORWAY

## Abstract

**Background:**

The use of evidence-based practice among healthcare professionals directly correlates to better outcomes for patients and higher professional satisfaction. Translating knowledge in practice and mobilizing evidence-based clinical care remains a continuing challenge in healthcare systems across the world.

**Purpose:**

To describe experiences from the implementation of an Early Detection and Treatment Program for Children (EDT-C) among health care professionals at a pediatric hospital in Sweden.

**Design and methods:**

Sixteen individual interviews were conducted with physicians, nurses and nurse assistants, which of five were instructors. Data were analyzed with qualitative content analysis.

**Results:**

An overarching theme was created: From uncertainty and skepticism towards assurance and control. The theme was based on the content of eight categories: An innovation suitable for clinical practice, Differing conditions for change, Lack of organizational slack, Complex situations, A pragmatic implementation strategy, Delegated responsibility, Experiences of control and Successful implementation.

**Conclusions:**

Successful implementation was achieved when initial skepticism among staff was changed into acceptance and using EDT-C had become routine in their daily work. Inter-professional education including material from authentic patient cases promotes knowledge about different professions and can strengthen teamwork. EDT-C with evidenced-based material adapted to the context can give healthcare professionals a structured and objective tool with which to assess and treat patients, giving them a sense of control and assurance.

## Background

Recognition and treatment of children developing critical illness is fundamental in hospital care, and hospital systems need to be developed to detect deterioration of patients´ health status at an early stage [1]. Unrecognized clinical deterioration in pediatric wards can lead to serious adverse events, unplanned intensive care and even cardiopulmonary arrest and unexpected death [2]. Prevention is dependent on the timely identification, referral and treatment [3]. The Pediatric Early Warning Score (PEWS) is a structured instrument for identification of patients at risk of deterioration, focusing on three components: behavior, color/cardiovascular status and respiratory status (scale from 0–9) [4–6]. It is a validated instrument for identifying patients vulnerable to acute deterioration in different pediatric settings such as general medicine, oncology and resource-limited settings [7–9]. PEWS was used as an initiative to increase patient safety at Uppsala University Hospital through the development of The Early Detection and Treatment Program for Children (EDT-C). In addition to PEWS, the EDT-C consists of four parts [10]: 1) Airway, Breathing, Disability, Exposure (ABCD), which is a structured tool to examine, treat and evaluate the patient´s vital functions on the basis of a predetermined order [11]: 2) Crew Resource Management (CRM), which is a model for teamwork acknowledging human error and human performance limitations, focusing on communication, situation awareness, leadership and resource management [12, 13]: 3) Situation, Background, Assessment, Recommendation (SBAR), which is a standardized tool for clear and concise communication [14]: 4) EDT- Ladder for Children, which is a clinical algorithm with recommended action according to the score in the assessed PEWS[10].

Furthermore, interdisciplinary working groups at each ward developed ward-specific guidelines. In March 2013, at The University Children’s Hospital, Uppsala, the EDT-C was introduced at three inpatient pediatric wards of the hospital trough an eight-hour training program for EDT-C instructors, in which a total of 12 instructors were trained. The instructors then trained staff on the ward to which they belonged. Inter-professional, mandatory three-hour training was conducted for all employees in the three wards where EDT-C was to be introduced [10]. The aim of the present study was to describe healthcare professionals’ experiences of the implementation process of EDT-C.

## Method

Interviews were conducted with healthcare professionals. The interviews took place between April and June 2013, which was about 2–3 months after the implementation start-up.

A purposive sampling technique was used, so as to obtain a mixed sample regarding experience and education. Participants received an information letter by email, stating the aim of the interviews. Furthermore, the letter stated that participation was voluntary, and that they would be free to withdraw from the interviews at any time, in line with the declaration of Helsinki [15]. The respondents gave verbal consent to attend. The study was approved by the Regional Ethical Review Board, Uppsala, Sweden (dnr 2012/407). The interviews took place in separate rooms within the hospital and were conducted by one physician (MM) and one pediatric nurse (GE). The interviews were recorded and notes were taken. Open-ended and follow-up questions regarding their experience of EDT-C education, materials and the implementation process were asked.

### Participants and setting

In total, 16 healthcare professionals, two men and 14 women were interviewed. Among participants, there were 11 nurses, 3 medical doctors and 2 nurse assistants. Five of the 16 participants interviewed had acted as instructors during the project implementation. The period of employment at the children’s hospital among the participants varied between 3 months and 34 years. All the participants worked at the University Children´s Hospital, Uppsala, and worked in one of three wards: the general pediatrics and emergency care ward, a ward for both planned and emergency care for neurology and surgery patients and finally, one ward for blood and tumor diseases.

### Data analysis

Data were analyzed using qualitative content analysis [16]. The first author listened to and read all transcripts to get a sense of the material as a whole. During the preparation phase the data were first analyzed using an inductive approach [17, 18]. Meaning units that responded to the aim were identified and condensed [19]. The condensed meaning units were then grouped using a deductive approach [17, 18] with data organized according to the five dimensions of i-PARIHS (integrated–Promoting Action on Research Implementation in Health Service): Innovation, Recipients, Context, Facilitation, and Implementation outcomes. The i-PARIHS framework is widely used when studying implementation of evidence into practice [20]. Thereafter preliminary categories and subcategories within the five dimensions were identified. During this process there was an ongoing discussion and reflection between the authors. Finally, categories and subcategories were formulated. From the contents of the categories and subcategories an overarching theme was created.

## Results

The combined inductive and deductive process resulted in eight categories and 17 subcategories (Table 1). An overarching theme was created based on the respondents’ descriptions of the implementation process: From uncertainty and skepticism towards assurance and control. The theme captures both the internal process of individual staff members as well as the cultural shift that was seen in relation to EDT-C. In the following sections categories and sub-categories are presented. Quotations from informants are labelled with a number and their profession; Physician (P), Registered Nurse (RN) and Nurse Assistant (NA). It is also indicated if they were instructors (I).

**Table 1:** Categories and subcategories describing the healthcare professionals’ and instructors’ experiences of the EDT-C program.

Categories within the dimensions	Subcategories
***Innovation***	
An innovation suitable for clinical practice	An objective and structured instrument
	An ambiguous instrument
***Recipients***	
Differing conditions for change	Positive attitudes towards new routines
	Skepticism towards new routines
	Differing knowledge among staff
***Context***	
Lack of organizational slack	Lack of time and personnel resources
Complex situations	Heterogeneous patient group
	High level of specialization
***Facilitation***	
A pragmatic implementation strategy	Interprofessional training
	Realistic and clear training
Delegated responsibility	Ward-specific guidelines
	Stakeholder support
***Implementation outcomes***	
Experiences of control	Perceptions of increased patient safety
	Increased level of confidence
	Changes in motivation
	Perceptions of improved communication
Successful implementation	Innovation as routine

### An innovation suitable for clinical practice

Participants perceived the training material as clear and readily applicable to clinical practice. PEWS was considered to be an objective, structured instrument, although it included elements of subjective assessment.

#### An objective and structured instrument

To learn a structured way to examine and treat the patient was experienced as positive. Participants perceived that PEWS was an objective way to assess the child's vital parameters.”*You understood why one wanted to introduce PEWS*, *that you can detect changes much earlier with objective parameters*.*” (HP-P1)*. Some healthcare professionals perceived that PEWS facilitated communication about the patient’s health status by using an objective and structured instrument.

#### An ambiguous instrument

Both instructors and healthcare professionals however described some difficulties in assessing PEWS in the start-up phase. Uncertainties were observed in measuring capillary refill/ pallor and assessing the degree of alertness and they stated that they needed to discuss with each other around these parameters;*”Some things that you assess are more subjective and that requires a somewhat more experienced assessor” (HP-P1)*.

### Differing conditions for change

Participants described both positive and skeptical attitudes when first introduced to EDT-C.

#### Positive attitudes towards new routines

The fact that new routines and instruments were often introduced were perceived as stressful; there was constant pressure to learn new things. On the other hand, some described how they believed that EDT-C could make a positive contribution to their work situation. They saw a practical benefit with the concept since it gave them a tool to work with;”*I thought it seemed good*, *and then I like to teach and it felt fun*, *a good project*, *so to say* …*” (I-P3)*. Participants stated that clear guidelines for how and what to implement, and a good motivation concerning why, is necessary for the easier adoption of new guidelines and methods.

#### Skepticism towards new routine

However, some participants indicated that it was important to be critical because not all new methods do lead to an improvement;”*You're always skeptical about what’s new and sometimes it’s justified*, *so you always need to evaluate and consider” (HP-P1)*. One participant described how attitudes towards new routines differed depending on level of experience. As an experienced healthcare professional you feel safe and comfortable with the old routines. Whereas when less experienced you feel some insecurity with old routines and more easily and willingly embrace new things that you hope improve practice;”*If you’ve been working for a long time*, *you’ve learnt your routines and you think it’s good to know that they work*, *so why should you bring in new ones*?*” (HP-RN5)*.

#### Differing knowledge among staff

In the process to formulate ward-specific guidelines, the healthcare professionals and instructors described that they were to a large extent dependent on each other's knowledge areas and the higher medical knowledge of the physicians. The interprofessional perspective was emphasized, with a clear allocation of work and roles within the team;”*In our ward we work a lot in teams where everyone’s voice is very valuable*. *Everyone works from their perspective and* … *I think that’s necessary*, *because we’ve got severly sick children” (HP-P2)*.

### Lack of organizational slack

All the participants described lack of time and personnel resources as the biggest obstacle to educating, supporting and developing EDT-C in an optimal way.

#### Lack of time and personnel resources

Lack of time resulted in brief training sessions for physicians, who often felt under pressure and had to cancel patients in order to participate. A high workload sometimes made it impossible to carry out training programs as planned;”*Even though we had booked the sessions in*, *the staff couldn’t get away because of the high workload*, *someone has to work” (I-RN2)*.

One of the instructors felt it difficult to take on the role of facilitator after the training since they were not freed from their regular duties and the work schedule had not been adapted.

### Complex situations

Participants described how the complexity of the patient groups and the different specialization areas within each of the three wards posed a challenge to the introduction of EDT-C.

#### Heterogeneous patient group

Participants described that patient groups varied, there were those who had an underlying condition and a majority that did not. The former group was especially tricky since they many times fell outside the reference values in their normal state. This made it difficult to assess their condition using the standard tools;”*You have to assume that the children who are basically healthy have normal reference ranges*, *but we have a lot of children who do not have normal reference ranges from the outset” (HP-RN3)*.

#### High level of specialization

The participants pointed out that in a unit with several different medical areas it became very complex to formulate guidelines and carry out training;”*We have discussed guidelines with the doctor in the group*. *There are four different specialist areas and*, *of course*, *very different patients (I-RN1)*.

### A pragmatic implementation strategy

To have an interprofessional approach was considered important in order to gain an understanding of each other’s professions, and that while working towards the same goal, one focuses on different things.

#### Interprofessional training

It was considered important that the instruction and training was led by both nurses and physicians since this was an interprofessional routine being introduced;”*EDT-C is good for this to include all the (staff) categories*. *There were both nurses and doctors in this group in the department appointed as trainers*. *That was really good” (HP-RN2)*

#### Realistic and clear training

Both instructors and healthcare professionals described that the practical exercises with patient cases provided an increased understanding of why and how to use the different parts of EDT-C; *“Patient cases*, *that was also good because then you could see a little more clearly how it could be good to use PEWS earlier in that specific patient case” (HP-NA2)*. Some stated that it was important for everyone to feel involved in the training. The participants recounted that they felt the training led to structured teamwork and that this structure in turn led to increased patient safety. Instructors described that they experienced the instructor’s training as clear and structured, and concluded that they had tried out a good way to teach together.

Reminders in the form of small pocket-sized sheet with PEWS, reference values and SBAR that was handed out were perceived as useful when checking patient parameters. Another facilitating factor that was appreciated was posters with the EDT Ladder for Children placed in strategic places on the wards as reminders in the daily work.

### Delegated responsibility

The development of ward-specific guidelines that were clear and context-specific was considered important for the successful implementation of EDT-C.

#### Ward-specific guidelines

One participant stated that it was not until the guidelines were drafted that the introduction of EDT-C went smoothly. Furthermore, clear medical prescription as to whether or not PEWS was to be assessed was considered significant in order not to lose motivation. One instructor even suggested to have more scientific background facts in the training material and that the training should have been more focused on how to teach as an instructor.

#### Stakeholder support

Participants perceived that they could ask instructors if they had questions about EDT-C, and the instructors felt that this was included in their task. Several also described their role as instructors as that of driving and encouraging their employees in the department, acting as a stakeholder;”*[As an instructor] you were expected to be present in the ward*, *encouraging so to say*, *and also to be a little inspiring too” (I-RN1)*.

### Experiences of control

The EDT-C program was perceived as a support in the care of children and gave a sense of control.

#### Perceptions of increased patient safety

Participants perceived an increased patient safety, both through an earlier detection of deterioration and through a deepened understanding about what was actually assessed and what it meant for the patient’s state of health;”*I think you have better control actually*. *In seeing a change*. *You think about that a bit more perhaps*. *A little extra” (HP-NA1)*.

#### Increased level of confidence

Particularly less experienced healthcare professionals perceived PEWS as a security that could guide them in all age-specific reference ranges related to pediatric care. This led to increased self-confidence;”*It will be both a reassurance for the patient*, *but also for me as* … *a member of staff*, *the possibility to easily make an assessment of the patient” (I-RN1)*. It was also stated that physicians perceived it to increase their sense of patient security when knowing that controls were carried out routinely even if it was not actively prescribed. To be able to make, what healthcare professionals perceived as an objective assessment, was described as significant and that it often strengthened them in their work.

#### Changes in motivation

After the ward-specific guidelines had been developed, the participants also expressed more motivation to use PEWS and that their understanding of the instrument had increased. However, the knowledge and the willingness to use EDT-C as a concept varied, and most diversity was observed in the physicians’ group. In this group, concerns emerged during the training sessions about increased workload in terms of more calls from the wards. Several instructors felt that the introduction to the different wards had worked relatively smoothly even though there were still many parts that needed to be developed. Discussion in the physicians’ group around the estimated increased workload decreased and it was noted that the workload had actually not increased;”*It was probably the fear that there would be a lot of extra work*, *but it’s not my experience at all that it’s led to a heavier workload and absolutely not an unnecessary workload” (HP-P1)*.

#### Perception of improved communication

The physicians stated that when communicating by telephone, the situation was more clearly described when using the tool;”*I think it facilitates communication and I think EDT-C is good” (I-P1)*. Nurses felt that physicians were more likely to take a stand on the PEWS reported, and physicians felt that they got more clear description of the patient’s condition when receiving reports over the phone or at ward rounds.

### Successful implementation

Participants stated that EDT-C was now used in their daily work routine, and that it created a more structured way of working.

#### Innovation as routine

Measuring PEWS had become a routine, leading to increased reflection on the patient’s health condition;”*It’s quite simply a routine now*” (I-RN2). PEWS had become a way to secure assessments made by being able to go back and check how PEWS has evolved over time. It was easier to follow a process of deterioration or improvement with PEWS;”*You still get a wake-up call*, *is this child getting worse now when PEWS is rising*” (HP-RN6). It was stated that the objective assessment, which was not influenced by personal characteristics or experience, was essential both for the patient and for the healthcare professionals.

## Discussion

Results reveal that despite initial hesitance towards changes in work routines, healthcare professionals managed to embrace and implement EDT-C. The program was perceived as providing a structured tool, even if it was sometimes considered ambiguous and requiring other skills and routines to support it. Initially, participants expressed skepticism as well as some positive responses towards the introduction of new routines when working within complex care situations. A pragmatic implementation strategy, interprofessional training and delegated responsibility facilitated the healthcare staff to familiarize with the EDT-C program and to be convinced about its advantages, despite a lack of organizational slack. The EDT-C program and specifically the use of PEWS, aided staff to gain a sense of assurance and control as it became a routine tool to assess health status among patients. This process was thus understood and expressed by the theme: From uncertainty and skepticism towards assurance and control.

The immediate local context and the wider organizational context influence all implementation processes [20]. In this case, mixed attitudes towards the new routine were apparent. In a complex organization with ongoing adaptions to new methods, learning new things may invoke negative attitudes, and recipients have varying levels of ability to adopt an innovation [21], as was described by respondents in the present study. Furthermore, according to the respondents, lack of time and personnel resources was an obstacle in the implementation process and caused frustration. Implementation of methods that are evidence based and have a clear clinical relevance will probably also have the potential to reduce skepticism and promote willingness to make an effort in a slimmed organization. In the present project, leaders at all levels supported the implementation of EDT-C. Nevertheless, managers and the project leaders need to be prepared and ready for emerging problems in order to handle unforeseen situations.

The respondents indicated that interprofessional education increased their understanding of different professionals’ specific knowledge and approaches, something that was positive for the teamwork. This is supported by earlier findings indicating that different professions learn together with, from and about each other [22]. Further, it was of significance that both physicians and nurses led the education, since interprofessional teamwork is required to manage the complexity of children’s care [23]. It has also been reported that interprofessional education is essential as a part of a targeted strategy to promote patient safety in pediatric care [2]. In a similar initiative to increase patient safety at a pediatric hospital in Norway investigating barriers and enabling factors for implementation, positive feedback on performance was pointed out as an important enabler when introducing new routines [24]. This was not brought up in this study, but is worth highlighting since research tends to focus on barriers rather than on enabling factors.

### Methodological consideration

Implementation of multi-facetted interventions in complex settings is influenced by a variety of factors. A structured framework can facilitate understanding of the dynamic nature of implementation [20, 25]. In this study, PARiHS was used in the implementation process and i-PARiHS for the evaluation, which strengthens both the implementation phase and the evaluation phase [25, 26].

We initially analysed the material with an inductive approach [27] instead of directly applying the i-PARiHS framework. This secured that all data in the material relating to the objective were captured. With the subsequent deductive approach, grouping all condensed meaning units according to the theoretical framework ensured that all dimensions of the implementation process were investigated [17, 26]. Credibility was strengthened by the use of the same open questions during all interviews so that the same area was covered. Dependability was ensured by the fact that one author analysed the data and then discussed the findings with all the other authors on several occasions, by maintaining similarities within, and differences between the categories, and further through a clear description of the analysis process. Conformability was strengthened by the result being based on the material as shown by quotes. Transferability was promoted by a clear and distinct description of the sampling procedure, the culture and context and by including participants with different degrees of education.

There are some limitations to the study worth mentioning. The risk of desirability bias is always present in embedded research and cannot be ignored in this study. However, the study subjects were in no dependency situation towards the researcher and an open and democratic culture was promoted, reducing the risk of bias. Embedded research, with all investigators being familiar to the project may also have influenced the interpretation of the results. An open mind set minimizes bias from the pre-knowledge and a reflective approach was pursued during the process until consensus was reached. Finally, the limited number of interviews can raise doubts about the generalizability of results. However, the selection of participants was done openly by others than the research team to ensure that all opinions were represented. Furthermore, saturation was achieved in the material, covering both positive and negative aspects and attitudes.

## Conclusions

Successful implementation was achieved when initial skepticism among staff was changed into acceptance and using EDT-C had become routine in their daily work. Inter-professional education including material from authentic patient cases promotes knowledge about different professions and can strengthen teamwork. EDT-C with evidenced-based material adapted to the context can give healthcare professionals a structured and objective tool with which to assess and treat patients, giving them a sense of control and assurance.
